# Transcriptomic and Extracellular Proteomic Analyses Implicate Two-Component Signaling in Medium-Dependent Virulence in *Vibrio mediterranei*

**DOI:** 10.3390/vetsci13101069

**Published:** 2026-10-10

**Authors:** Haiyan Zhang, Yihan Luo, Xiaohui Zou, Sheng Liu, Zhihua Lin, Qinggang Xue

**Affiliations:** 1 College of Advanced Agricultural Science, Zhejiang Wanli University, Ningbo 315100, China; haiyan.zhang@zwu.edu.cn (H.Z.);; 2 Zhejiang Key Laboratory of Aquatic Germplasm, Zhejiang Wanli University, Ningbo 315100, China; 3 Ninghai Institute of Mariculture Breeding and Seed Industry, Zhejiang Wanli University, Ningbo 315604, China

**Keywords:** *Vibrio mediterranei*, pathogenicity, shellfish, ECPs, transcriptomic analysis

## Abstract

**Simple Summary:**

*Vibrio mediterranei* is associated with vibriosis in cultured bivalves, yet how environmental conditions govern its virulence remains unrevealed. We compared the pathogenicity of *V. mediterranei* strains toward razor clam (*Sinonovacula constricta*) juveniles after growth in 2216E and TCBS media. TCBS-grown bacteria caused up to 98.9% juvenile mortality, whereas 2216E-grown bacteria induced less mortality. Profiling of extracellular proteins identified 646 and 399 differentially expressed proteins in DT07 and DT02, with enrichment in secretion-related and cell envelope processes and in the two-component system, and in the two-component system, while RNA sequencing revealed 3276 and 2084 differentially expressed genes, dominated by carbon metabolism, flagellar assembly, membrane transport, and signal transduction. Linking both datasets through KEGG Orthology identifiers pointed to the two-component system (ko02020) as the most prominently represented shared pathway, with *fliC*, *tolC* and *crp* coupling transcriptional change to extracellular protein abundance. These results indicate that medium composition shifts the virulence of *V. mediterranei* in association with two-component signaling, associated with motility, extracellular protein abundance, and metabolic adaptation, and provides a candidate target for functional validation in bivalve aquaculture.

**Abstract:**

*Vibrio mediterranei* is an emerging pathogen causing vibriosis outbreaks in marine bivalves, yet the molecular mechanisms by which environmental conditions modulate its virulence remain poorly understood. Here, we combined in vivo infection assays with integrated transcriptomic and extracellular products (ECPs) proteomic analyses to dissect how culture medium composition shapes the virulence of *V. mediterranei* toward razor clam (*Sinonovacula constricta*) juveniles. All tested strains caused markedly elevated juvenile mortality when cultured in TCBS medium (up to 98.9%, *p* < 0.0001), whereas strains grown in 2216E caused low mortality (0–3.3%), and no mortalities were recorded in the bacteria-free seawater control. Comparative ECPs proteomics identified 646 and 399 differentially expressed proteins (DEPs) in DT07 and DT02, respectively, enriched in cell envelope-related processes and in the composition of the extracellular protein fraction, the two-component system (TCS), and oxidative pathways, while transcriptomics revealed 3276 and 2084 differentially expressed genes (DEGs) dominated by carbon and energy metabolism, flagellar assembly, membrane transport, and signal transduction. Cross-omics integration via KEGG Orthology identifiers identified that the TCS (ko02020) was among the most prominent pathways linking the transcriptomic and ECPs proteomic layers, with *glnA*, *pstS*, *crp*, *fliC*, and *tolC* detected at both levels. *fliC*, *tolC* and *crp* were consistently detected at both two layers, linking transcriptional reprogramming to extracellular protein abundance. These findings implicated two-component signaling in the increased virulence of *V. mediterranei* in TCBS medium associated with motility, extracellular abundance, and metabolic adaptation, providing a framework for hypothesis-driven validation linking environmental cues to pathogenic potential in shellfish-associated *Vibrios*.

## 1. Introduction

*Vibrio mediterranei* belongs to the Mediterranean clade of the family Vibrionaceae and was first isolated from plankton, sediment, and seawater at two coastal sites of Valencia, Spain, in 1986 [1]. Since then, *V. mediterranei* has been reported worldwide in association with a broad range of marine organisms, including invertebrates [2,3,4,5,6], fish [7,8], and algae [9], and has been identified as a causative agent of bivalve vibriosis, coral bleaching, and yellow spot disease of *Pyropia*. The pathogenicity of *V. mediterranei* has been linked to quorum-sensing mechanisms mediated by signaling molecules such as N-acylhomoserine lactones (AHLs), bacterial adhesion, superoxide dismutase production, and toxin production [2,6,10,11]. Genomic analyses of *V. mediterranei* have identified virulence-associated genes encoding toxin-coregulated pilus (TCP), superoxide dismutase (SOD), repeats-in-toxin (RTX), melanocyte-stimulating hormone (MSH), and zonula occludens toxin (ZOT) [12].

The pathogenicity of *Vibrio* species is not a fixed trait but is dynamically shaped by the surrounding environment. Temperature, osmotic stress, pH, and nutrient composition have all been reported to modify the expression of motility, secretion, adhesion, and toxin genes in multiple *Vibrios* [13,14]. RNA-seq profiling of *V. parahaemolyticus* revealed that temperature and nutrition reprogram the transcriptome that measurably increases shrimp mortality [15], while exposure to low salinity mobilizes the type III secretion system of the same species [16]. Bile salts, a special component of animals, have been identified as direct chemical inducers of two-component signaling pathways in *V. cholerae*, where they activate the EnvZ/OmpR system to modulate virulence factor expression [17,18].

The virulence of *V. mediterranei* is also affected by environmental factors. *V. mediterranei* begins to exhibit pathogenicity toward corals at 24 °C, with virulence increasing progressively as temperature rises and reaching a maximum at 28 °C [19]. As a pathogen of juvenile bivalves, *V. mediterranei* cultured in TCBS medium displays significantly increased pathogenicity toward juvenile razor clams (*Sinonovacula constricta*) than that cultured in 2216E medium, and extracellular protein profiles under TCBS conditions are more complex than those under 2216E conditions [6]. 2216E, a nutrient-rich marine formulation, and TCBS, a selective medium that imposes bile-salt and alkaline pH stress [20], represent two ecologically distinct nutritional regimes. Characterizing the expression patterns of these virulence factors under different environmental conditions will provide an important cue for the prevention and control of *V. mediterranei*. Yet the transcriptional and post-transcriptional response linking medium composition to virulence remains undefined.

To resolve these responses, high-resolution analytical platforms are required. Strand-specific RNA sequencing captures genome-wide transcriptional reprogramming events [21,22], while tandem mass spectrometry-based proteomics of extracellular products (ECPs) reports the abundance of proteins recovered in the extracellular fraction [23]. Overlaying these two methods through shared KEGG Orthology (KO) identifiers enables systematic identification of pathways co-regulated at the mRNA and protein levels [24,25]. In signaling between environmental sensing and transcriptional expression, two-component systems (TCSs) perform an important role [26,27]. In *Vibrio* species, individual TCSs including EnvZ/OmpR [17], PhoR/PhoB [28], and CpxA/CpxR [29] together with the cAMP receptor protein (CRP), which couples carbon-source availability to virulence gene expression [30,31], have been functionally connected to the pathogenic phenotypes. Whether TCS-associated and CRP-dependent pathways transduce medium-derived signals into virulence expression in *V. mediterranei* is unknown.

To date, no study has systematically integrated phenotypic virulence with transcriptomic and ECPs proteomic analyses to reveal the molecular pathway from medium composition to virulence of *V. mediterranei*. In the present study, we addressed these knowledge gaps through an integrated experimental strategy combining in vivo virulence assays using juvenile razor clams, comparative transcriptomic profiling, ECPs proteomic analysis, and omics integration employing KO identifiers as the bridge. Two *V. mediterranei* strains with contrasting virulence, DT02 (low virulence) and DT07 (high virulence), were cultured in 2216E and TCBS media, and phenotypic, transcriptomic, and proteomic comparisons were performed to systematically characterize medium-dependent virulence regulation.

## 2. Materials and Methods

### 2.1. Bacterial Strains and Animals

The *Vibrio mediterranei* strains DT01, DT02, DT03, DT07, NBSC231102, NBSC231104, and NBSC231105, together with *Pseudoalteromonas nigrifaciens* and *V. parahaemolyticus*, were cultured as previously reported [6,32]. All strains were cultured in 2216E or TCBS liquid medium at 28 °C with 200 rpm or on 1.5% (*w*/*v*) agar plates at 28 °C under inverted incubation. For all virulence assays and trascriptomic sampling, broth culture was incubated at 28 °C with shaking at 200 rpm for 12 h and harvested in the late logarithmic phase (OD600 = 0.5–0.7). Extracellular products for proteomic analysis were instead recovered from agar plates by the cellophane plate technique described in Section 2.3. TCBS was used in both forms, as agar plates for ECPs preparation and as liquid medium (peptone 10.0 g, yeast extract 5.0 g, sucrose 20.0 g, sodium thiosulfate 10.0 g, sodium citrate 10.0 g, bile salt 5.0 g, sodium cholate 3.0 g, NaCl 10.0 g, ferric citrate 1.0 g, bromothymol blue 0.04 g, thymol blue 0.04 g per liter; pH = 8.6 ± 0.2) (HB4130-1, HOPEBIO, Qingdao, China) was used only in liquid condition, and 2216E (peptone 5.0 g, yeast extract 1.0 g, ferric citrate 0.1 g, NaCl 19.45 g, MgCl2 5.98 g, Na_2_SO_4_ 3.24 g, CaCl_2_ 1.8 g, KCl 0.55 g, Na_2_CO_3_ 0.16 g, KBr 0.08 g, SrCl_2_ 0.034 g, H_3_BO_3_ 0.022 g, Na_2_SiO_3_ 0.004 g, NaF 0.0024 g, NH_4_NO_3_ 0.0016 g, Na_2_HPO_4_ 0.008 g per liter; pH = 7.6 ± 0.2) (HB0132-1, HOPEBIO, Qingdao, China) for agar plate or liquid conditions. For infection assays, juvenile razor clams (*Sinonovacula constricta*) (4–6 mm) obtained from the breeding stock maintained at the Ninghai Institute of Mariculture Breeding and Seed Industry, Zhejiang Wanli University, China, were acclimated for 7 days in sterile artificial seawater (salinity 20 ± 1‰) at 22 °C with continuous aeration. During acclimation, seawater was replaced, and *Spirulina* powder was supplied daily.

### 2.2. Pathogenicity Analysis of Bacteria in Different Media

The strains described in Section 2.1 were recovered from the −80 °C freezer. Bacteria were harvested by centrifugation, followed by three washes with sterile seawater, and centrifuged at 4000 rpm for 5 min to remove extracellular products. The bacterial pellets were suspended in sterile seawater. Juvenile razor clams were divided into 6 × 6 × 6 cm containers, with 30 individuals and 20 mL of contaminated seawater per container. Each challenge was performed in triplicate. The final bacterial concentration was adjusted to 10^7^ CFU/mL based on absorbance at 600 nm (OD_600_), using a single OD600-to-CFU calibration applied to both media (an OD600 = 1.0 with the concentration at 10^9^ CFU/mL) as described [6]. The exposure dose was estimated by absorbance at OD_600_ rather than confirmed by plate counting, and a single OD_600_-to-CFU calibration was applied to both media. The concentration given throughout should therefore be read as nominal. The seawater was replaced daily to mimic the natural environment. The seawater without bacteria was set as the negative control. Mortality of razor clams was recorded at 6, 12, 24, and 36 h post-exposure. Each challenge thus comprised three parallel containers per condition (3 × 30 = 90 juveniles per condition).

### 2.3. ECPs Analysis

Extracellular products (ECPs) were prepared using the cellophane plate technique [33] with three biological replicates for each sample, as described in detail previously [6]. In brief, bacteria were spread on sterile cellophane membranes laid on the surface of TCBS and 2216E agar plates and incubated at 28 °C for 48 h, and cells were washed from the membrane with PBS every 6 h. Following vacuum concentration, ECPs underwent protein extraction, bicinchoninic acid (BCA) quantification, dithiothreitol (DTT) reduction, iodoacetamide (IAM) alkylation, and trypsin digestion. Protein quantification was performed by liquid chromatography-tandem mass spectrometry (LC-MS/MS) on an Ultimate RSLCnano 3000 ultra-performance liquid chromatography (UPLC) system coupled with a Q Exactive HF mass spectrometer. Raw data were searched against the UniProt *V. mediterranei* database (19,096 sequences) using MaxQuant (v.1.5.2.8) with label-free quantification (LFQ) and a false discovery rate (FDR) ≤ 0.01. Full details of sample preparation and instrument configuration have been reported previously [6]. The mass-spectrometry files analyzed here are the same batch generated for Fan et al. [6], where comparisons were made among strains rather than between media. In the present study, the raw dataset was re-analyzed along with the medium. All analysis results reported in this study are specific to the present study.

Differentially expressed proteins were defined as R > 1.3 (up-regulated) or R < 0.77 (down-regulated), where R is for the ratio of the mean LFQ intensity in TCBS to that in 2216E (three biological replicates per condition), with a Benjamini–Hochberg-adjusted *p*-value (FDR) < 0.05 (*t*-test). Because hundreds of proteins were tested simultaneously, *p*-values from the *t*-test were considered for multiple testing with the Benjamini–Hochberg procedure, and adjusted *p*-values were reported for every protein in Supplementary Table S4. Gene Ontology (GO) enrichment analysis was performed separately for the concordantly up-regulated (n = 169; 137 proteins with GO annotation) and down-regulated (n = 112; 43 with GO annotation) subsets of the shared DEPs using a hypergeometric test, with all identified proteins carrying GO annotation (n = 419) as the background. A total of 679 and 297 terms were tested for the up- and down-regulated subsets, respectively. Benjamini–Hochberg-adjusted *p*-values (FDR) were computed across all tested terms. The 20 most enriched terms for each sebset are chosen and illustrated, and the complete resulsincluding raw and adjusted *p*-values for every tested term, are provided in Supplementary Tables S2 and S3. Data analysis was performed by the sequencing company, and figures were generated using the Omicshare online platform (https://www.omicshare.com). KEGG pathway enrichment of the shared DEPs was performed using the same procedure against all identified proteins with KEGG annotation, and *p*-values were corrected using the Benjamini–Hochberg procedure.

### 2.4. Transcriptomic Analysis

Bacterial cultures for transcriptomic analysis were prepared from the same strains in the same two liquid media, under the liquid culture conditions given in Section 2.1 (28 °C, 200 rpm, 12 h). Three independent samples were included per group. Samples were flash-frozen in liquid nitrogen and stored at −80 °C and sent to Gene Denovo Biotechnology Co., Ltd. (Guangzhou, China) for sequencing and analysis. In brief, total RNA was extracted using the Trizol method, and RNA concentration, purity, and integrity (RIN) were assessed using a Nanodrop 2000 spectrophotometer (Thermo Fisher Scientific, Waltham, MA, USA) and an Agilent 2100 Bioanalyzer (Agilent Technologies, Santa Clara, CA, USA). Qualified samples were subjected to ribosomal RNA depletion using the Ribo-Zero rRNA Removal Kit (Illumina, San Diego, CA, USA), and strand-specific transcriptomic libraries were constructed using the NEBNext Ultra II Directional RNA Library Prep Kit for Illumina (NEB#7490, New England Biolabs, Ipswich, MA, USA). Library quality was evaluated using the Agilent 2100 Bioanalyzer and the ABI StepOnePlus Real-Time PCR System (Life Technologies, Frederick, MD, USA), followed by high-throughput sequencing on an Illumina NovaSeq X Plus (Illumina, USA) platform with 150 bp paired-end reads. Raw data were filtered using fastp (version 0.20.0) to remove reads containing adapters, reads with more than 10% ambiguous bases (N), and reads in which more than 50% of bases had a quality value ≤ 20; between 15.57 and 19.44 million clean reads were obtained per sample (96.45–99.54% of raw reads). Reads matching rRNA sequences, identified with Bowtie 2 (version 2.2.8), were removed, and remaining reads were aligned to the *V. mediterreanei* DT07 reference genome (GenBank accession JBHNVQ000000000.1) using Bowtie2. Alignment rates ranged from 87.03% to 93.15% for DT02 and from 97.91% to 98.96% for DT07 (Supplementary Table S1). Gene expression was quantified using Salmon (Version 1.10.3) and normalized to TPM. Differentially expressed genes (DEGs) were identified with edgeR package (version 3.12.1) from raw read counts, with |Log_2_ fold change| > 1 and FDR < 0.05. (*p-*value adjusted by the Benjamini–Hochberg method). Reproducibility among replicates was assessed using Pearson correlation coefficients (psych package, version 1.8.4) and principal component analysis (gmodels package). GO enrichment was tested by a hypergeometric test with Bonferroni correction (corrected *p* ≤ 0.05) and KEGG pathway enrichment by a hypergeometric test with FDR correction (*q*-value ≤ 0.05). Both analyses were conducted by the sequencing company, and figures were generated using the OmicShare online tool (https://www.omicshare.com/tools/).

Because DT02 and DT07 were quantified against a single reference (DT07), and DT02 reads mapped less efficiently to this reference than DT07 reads (84.6–91.8% vs. 94.5–95.6% unique mapping rates, Supplementary Table S1), cross-strain comparisons were restricted to genes detected in all 12 libraries. Since our aim was to identify common changes, DT02 genes absent from the DT07 reference genome were not included in the analysis. Thus, this mapping bias does not affect the results.

### 2.5. Integrated Analysis of ECPs and Transcriptomics

To identify virulence-related pathways differentially expressed in different media at both transcriptomic and proteomic levels, transcriptomic and ECP proteomic data were integrated into the analysis. Shared KO identifiers were detected to identify genes differentially expressed in proteomics and transcriptomics (“shared genes”), which were then mapped back to their respective KEGG pathways and ranked by shared gene count per pathway to determine the key pathways. Association data were organized in a three-column structure (source pathway-shared gene-target pathway), and a Sankey diagram was generated using the OmicShare online tool (https://www.omicshare.com/tools/Home/Soft/sankey) (accessed on 13 July 2026) to visualize the results. Because several loci of this draft genome carry the same KO identifier, correspondence between the two datasets was established at the level of the KO number rather than of the individual genomic locus, and every locus detected is reported separately with its own statistics in Supplementary Table S5.

### 2.6. Statistical Analysis

Survival curve analysis of bacterial virulence to razor clams was performed using GraphPad Prism 8. Kaplan–Meier survival curves (Figure S1) were compared using the Gehan–Breslow–Wilcoxon test to describe the mortality changes during the experimental period. Cumulative mortality at 36 h was calculated for each container from the 30 juveniles it contained. The mortality was analyzed by two-way ANOVA with medium and strain as factors, followed by Holm–Sidak’s multiple comparisons test of TCBS vs. 2216E with each strain. The bacterial free control was excluded from the multiple comparisons analysis as no mortality occurred in either medium. *p* < 0.01 was considered statistically significant.

### 2.7. GenAI Tools

During the preparation of this work, the authors used DeepSeek-V4-Pro to improve language skills. After using this tool, the authors reviewed and edited the content as needed and took full responsibility for the content of the publication.

## 3. Results

### 3.1. Virulence of Vibrio mediterranei to Clams Under Different Culture Medium

**Figure 1:**
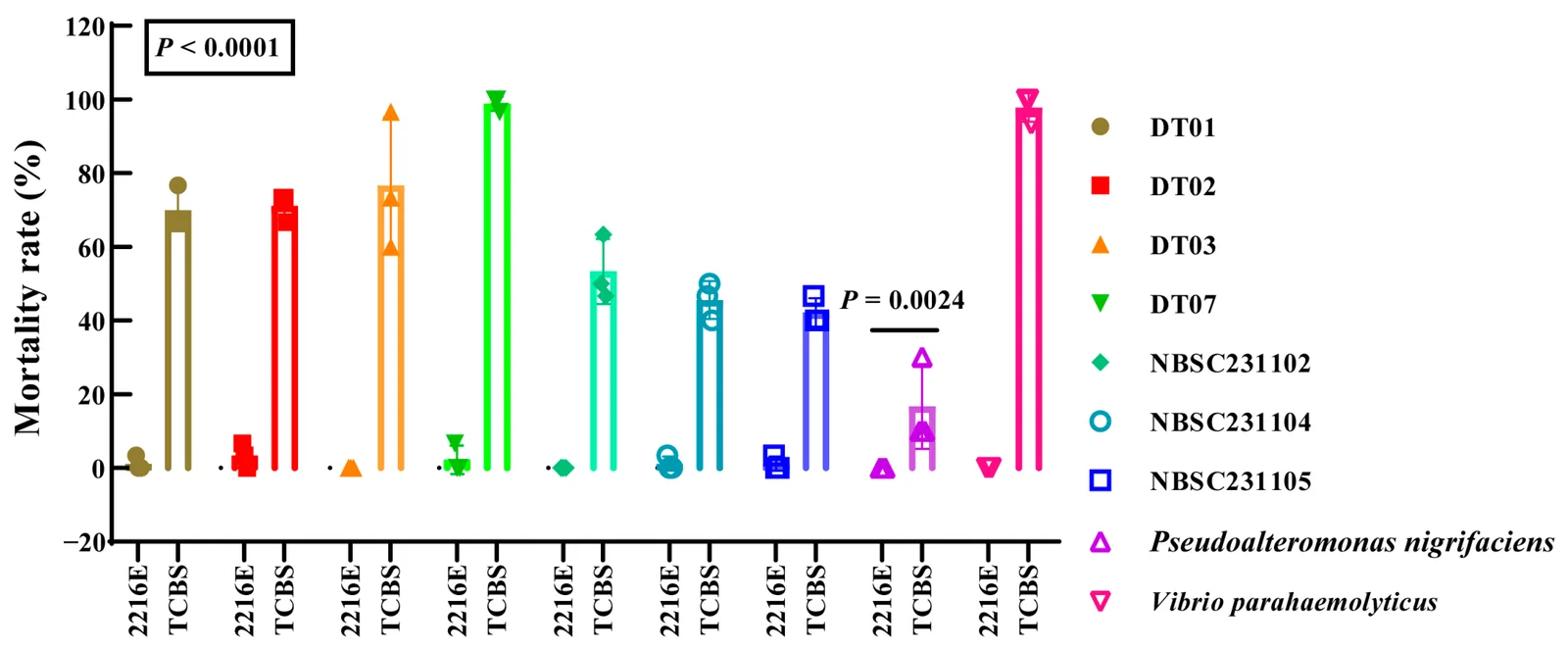
The mortality of razor clam juveniles exposed to bacteria cultured in different media. Bars show the cumulative mortality at 36 h for the nine bacterial strains grown in 2216E or TCBS (mean of three independent containers per condition, each containing 30 animals). Symbols show the value of each individual container, and error bars show SD. Bacterial-free controls (0% mortality in both media) are shown in Supplementary Table S6. Mortality was analyzed by two-way ANOVA with medium and strain as factors; the boxed *p*-value indicated the overall main effect of medium (*p* < 0.0001). Holm–Sidak’s multiple comparisons test of TCBS vs. 2216E with each strain showed *p* < 0.0001 for eight strains, except *p* = 0.0024 for *P. nigrifaciens*. *p* < 0.01 was considered significant.

To test the virulence of different *V. mediterranei* strains, *Pseudoalteromonas nigrifaciens,* and *V. parahaemolyticus* in razor clams, we performed exposure experiments of juveniles to artificial seawater containing bacteria with a final concentration at 10^7^ CFU/mL and recorded mortality. All tested bacteria, including all *V. mediterranei* strains, *P. nigrifaciens*, and *V. parahaemolyticus*, exhibited increased virulence toward razor clams when cultured in TCBS (Figure 1); mortality at 36 h was up to 98.89% for DT07 and 97.78% for *V. parahaemolyticus.* Whereas the same strains grown in 2216E caused little mortality (0–3.3% across the nine strains, 0% in the bacteria-free control). Two-way ANOVA showed a significant main effect of medium (*p* < 0.0001) together with an interaction (*p* < 0.0001). The Holm–Sidak’s multiple comparisons test confirmed higher mortality after juveniles’ exposure to TCBS-grown bacteria than to 2216E-cultured bacteria for every strain analyzed (*p* < 0.0001 except *P. nigrifaciens* with *p* = 0.0024) (Figure 1). Mortality also differed among strains grown in TCBS (one-way ANOVA, *p* < 0.0001) but not among strains grown in 2216E (*p* = 0.47).

### 3.2. The ECP Comparisons of V. mediterranei Cultured in Different Media

**Figure 2:**
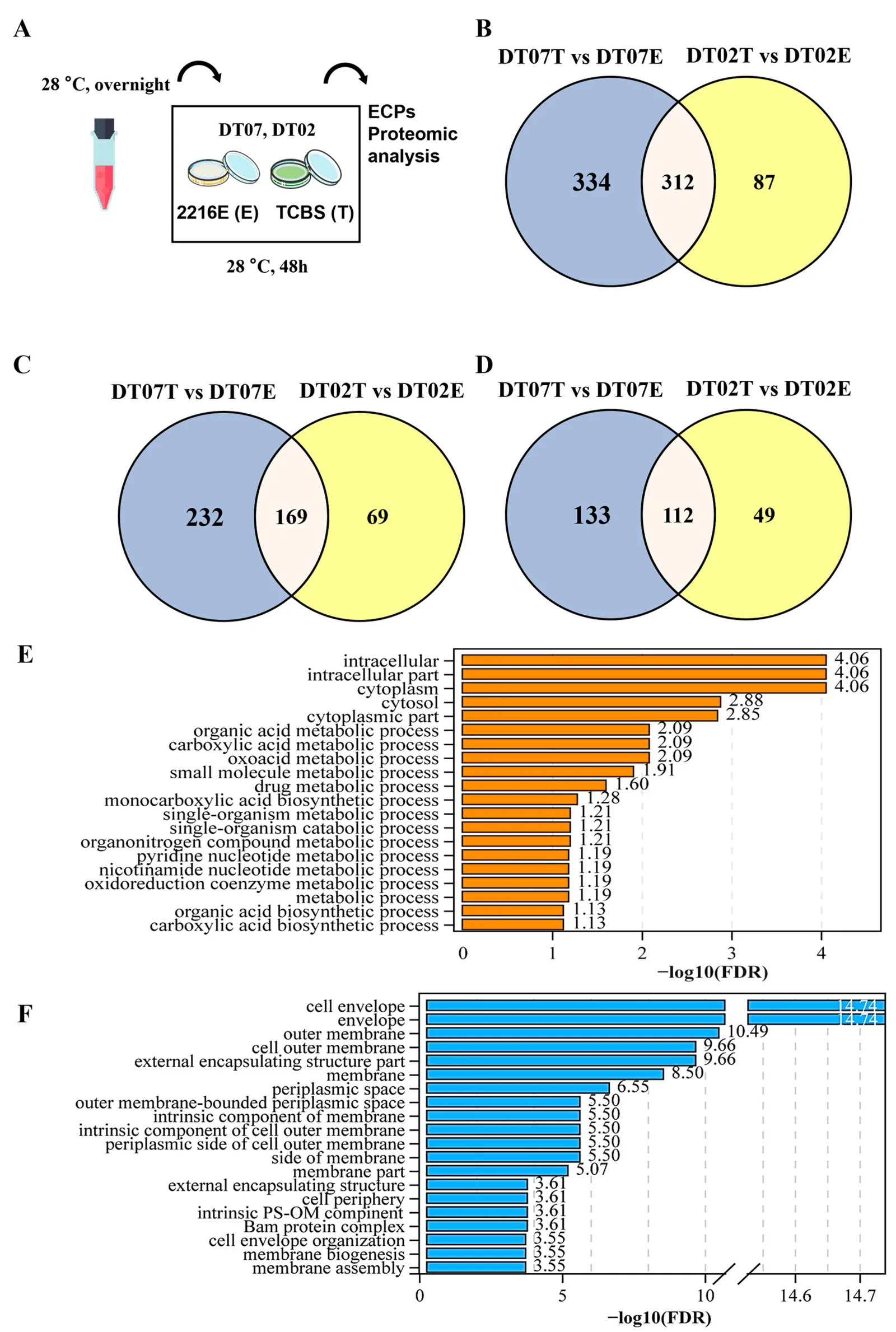
The ECPs’ analysis of *V. mediterranei* cultured in different media. (**A**) The flowchart of the experiments. (**B**) The Venn diagram of differentially expressed proteins between strains. (**C**) the up-regulated proteins of TCBS vs. 2216E between DT02T and DT07T. (**D**) the down-regulated proteins of TCBS vs. 2216E between DT02T and DT07T. (**E**,**F**) The GO enrichment analysis of the shared differentially abundant proteins. The concordantly up-regulated (E; n = 169) and concordantly down-regulated (F; n = 112) shared DEPs were tested for GO term enrichment by a hypergeometric test against all identified proteins with GO annotation (n = 419) as background, followed by Benjamini–Hochberg correction. The 20 most enriched terms are shown; bars represent −log10 (FDR). All terms shown in F and the ten highest-ranked terms in E have FDR < 0.05. PS-OM, periplasmic side of outer membrane; OM, outer membrane.

To compare the composition of ECPs from *V. mediterranei* under different media, proteomic analyses were performed with strains DT02 and DT07 cultured in 2216E (E) and TCBS (T) media. The differentially expressed proteins (DEPs) between T and E media (Figure 2B) analysis suggested 646 DEPs in DT07T vs. DT07E at FDR < 0.05 after BH correction, with 334 unique to DT07 and 312 shared; and 399 DEPs in DT02T vs. DT02E, with 87 unique to DT02 and 312 shared. These results indicate that TCBS medium substantially alters the ECP protein composition in both strains. The up-regulated and down-regulated ECPs of TCBS versus 2216E were further compared between the two strains (Figure 2C,D). Among up-regulated proteins, DT02T vs. DT02E and DT07T vs. DT07E shared 169 proteins, with 69 unique to DT02T vs. DT02E and 232 unique to DT07T vs. DT07E. Among the down-regulated ECPs, DT02T vs. DT02E and DT07T vs. DT07E shared 112 proteins, with49 unique to DT02T vs. DT02E and 133 unique to DT07T vs. DT07E. These findings suggest that TCBS medium alters the extracellular protein composition in a largely conserved way between strains, although some strain-specific differences remain.

GO and KEGG enrichment analyses were performed on the shared DEPs of DT02 and DT07, with *p*-values corrected for multiple testing using the Benjamini–Hochberg procedure. GO enrichment was tested separately for the concordantly up-regulated (n = 169) and down-regulated (n = 112) proteins (Figure 2E,F). The down-regulated proteins showed a highly focused response centered on the cell envelop (cell envelope, FDR = 1.8 × 10^−15^; outer membrane, 3.2 × 10^−11^; cell outer membrane, 2.2 × 10^−10^; periplasmic space, 2.8 × 10^−7^; Bam protein complex, 2.4 × 10^−4^), together with protein localization and transport terms, consistent with a reduced representation of outer-membrane and periplasmic proteins in the extracellular fraction. The up-regulated proteins were instead dominated by cytoplasmic localization and central carbon metabolism; among the 20 terms shown in Figure 2E, the ten highest-ranked were below FDR = 0.05 (cytoplasm, FDR = 8.7 × 10^−5^; cytosol, 1.3 × 10^−3^; carboxylic acid metabolic process, 8.2 × 10^−3^; small molecule metabolic process, 1.2 × 10^−2^). KEGG enrichment of the shared down-regulated proteins remained significant for ABC transporters (FDR = 1.9 × 10^−6^) and the two-component system (FDR = 0.022), whereas quorum sensing, NOD-like receptor signaling, plant–pathogen interaction, glutathione metabolism and prodigiosin biosynthesis, which were identified before correction, no longer reached significance and were not considered further. These results indicate that medium composition primarily modulates the composition of the extracellular proteome with respect to the envelope-associated proteins, which may be associated with the virulence of the strains toward shellfish. Complete results for every tested term are provided in Supplementary Tables S2 and S3.

### 3.3. The Transcriptomic Comparison of V. mediterranei Cultured in Different Media

**Figure 3:**
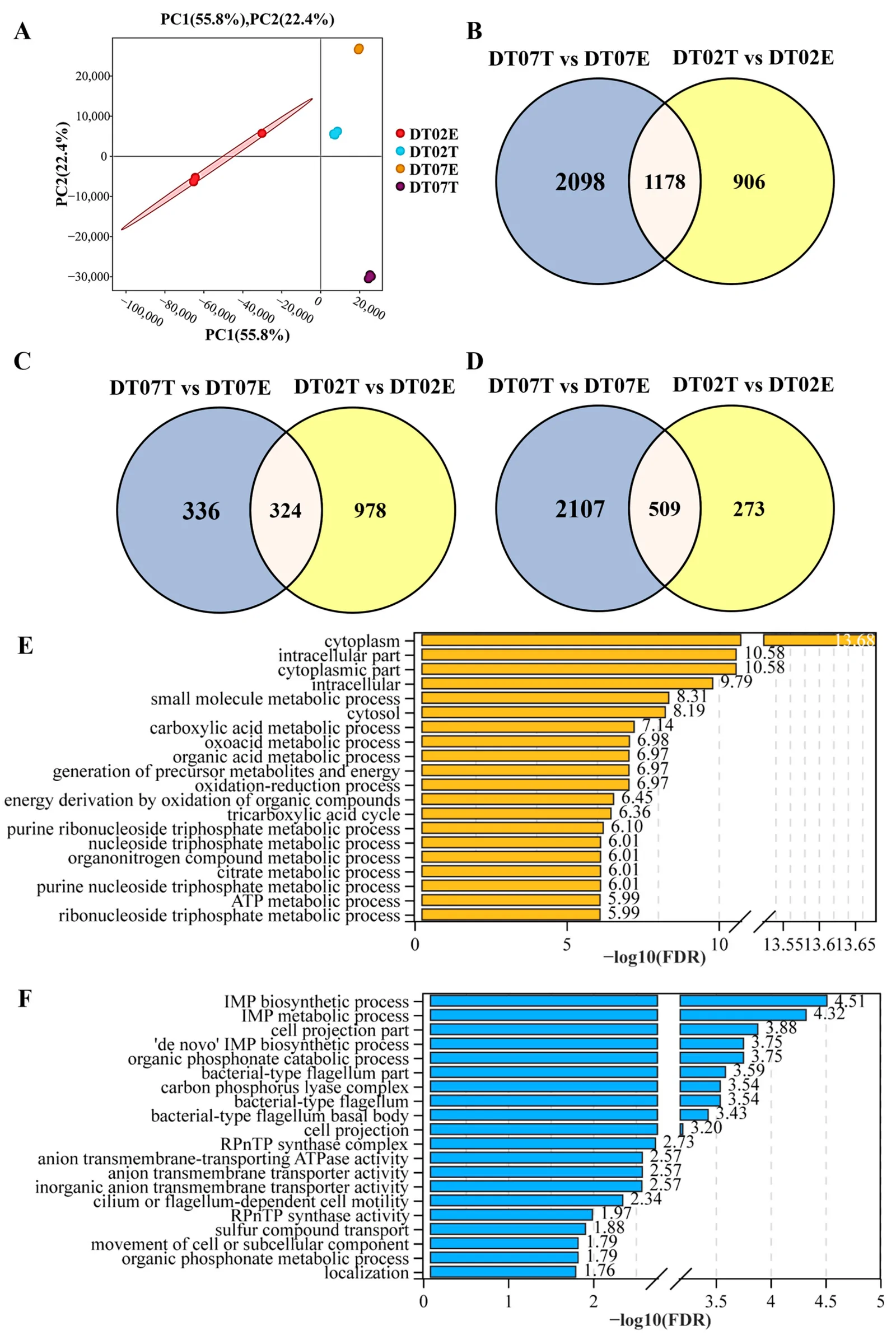
The transcriptomic analysis of *V. mediterranei* strains cultured in different media. (**A**) PCA analysis of the relationship between samples. (**B**) the Venn diagram of differentially expressed genes identified in DT02T vs. DT02E and DT07T vs. DT07E (|log2 fold change| > 1, FDR < 0.05). (**C**) Venn diagram of the up-regulated genes of TCBS vs. 2216E between the two medium contrasts. (**D**) Venn diagram of the down-regulated genes of TCBS vs. 2216E between the two medium contrasts. (**E**,**F**) Significantly enriched GO terms of the shared up-regulated (**E**) and shared down-regulated (**F**) DEGs, tested by a test against all genes with GO annotation as background, followed by Benjamini–Hochberg correction. RPnTP synthase, alpha-D-ribose 1-methylphosphonate 5-triphosphate synthase.

To understand the effects of the medium on the gene expression of *V. mediterranei*, we performed the transcriptomic analysis of DT02 and DT07 with the same treatments as those used in ECPs analysis. The PCA results (Figure 3A) showed that the first principal component (PC1, 55.8% of variance) completely separates DT02 from DT07, while the second principal component (PC2, 22.4% variance) further distinguishes medium 2216E from medium TCBS within each strain. This indicates that medium composition shows a distinct effect on gene expression in both strains.

Differential expression analysis (DEG) reveals differences in DEG counts across strains in different media. For the medium, DT07T vs. DT07E yielded 3276 DEGs (660 up-regulated, 2616 down-regulated), which is more than the 2084 DEGs (1302 up-regulated, 782 down-regulated) identified in DT02T vs. DT02E. This suggests that DT07 exhibits a stronger transcriptional response to medium change, and that its response is dominated by down-regulated genes. For the strain, DT07E vs. DT02E and DT07T vs. DT02T produced 2873 DEGs (2121 up-regulated, 752 down-regulated) and 3440 DEGs (1276 up-regulated, 2164 down-regulated), respectively. The larger number of DEGs between strains than in the medium shift within a strain, but these counts are not a formal test of the relative contribution and also cannot indicate that strain background has a greater impact on gene expression than medium composition.

The Venn diagram analysis of the DEGs (Figure 3B–D) further characterized the conserved and strain-specific components of the gene expression response to the medium. Of the 2084 and 3276 DEGs identified in DT02 and DT07, respectively, 1178 were shared between the two strains, with 906 unique to DT02 and 2098 unique to DT07 (Figure 3B). Among up-regulated genes (Figure 3C), the two strains shared 324 DEGs, with 978 genes uniquely up-regulated in DT02 and 336 genes uniquely up-regulated in DT07, indicating that DT02 activates more up-regulated genes. Among down-regulated genes (Figure 3D), a larger shared set of 509 DEGs was identified, with 2107 genes uniquely down-regulated in DT07 and 273 genes uniquely down-regulated in DT02, consistent with the stronger transcriptional repression observed in DT07.

**Figure 4:**
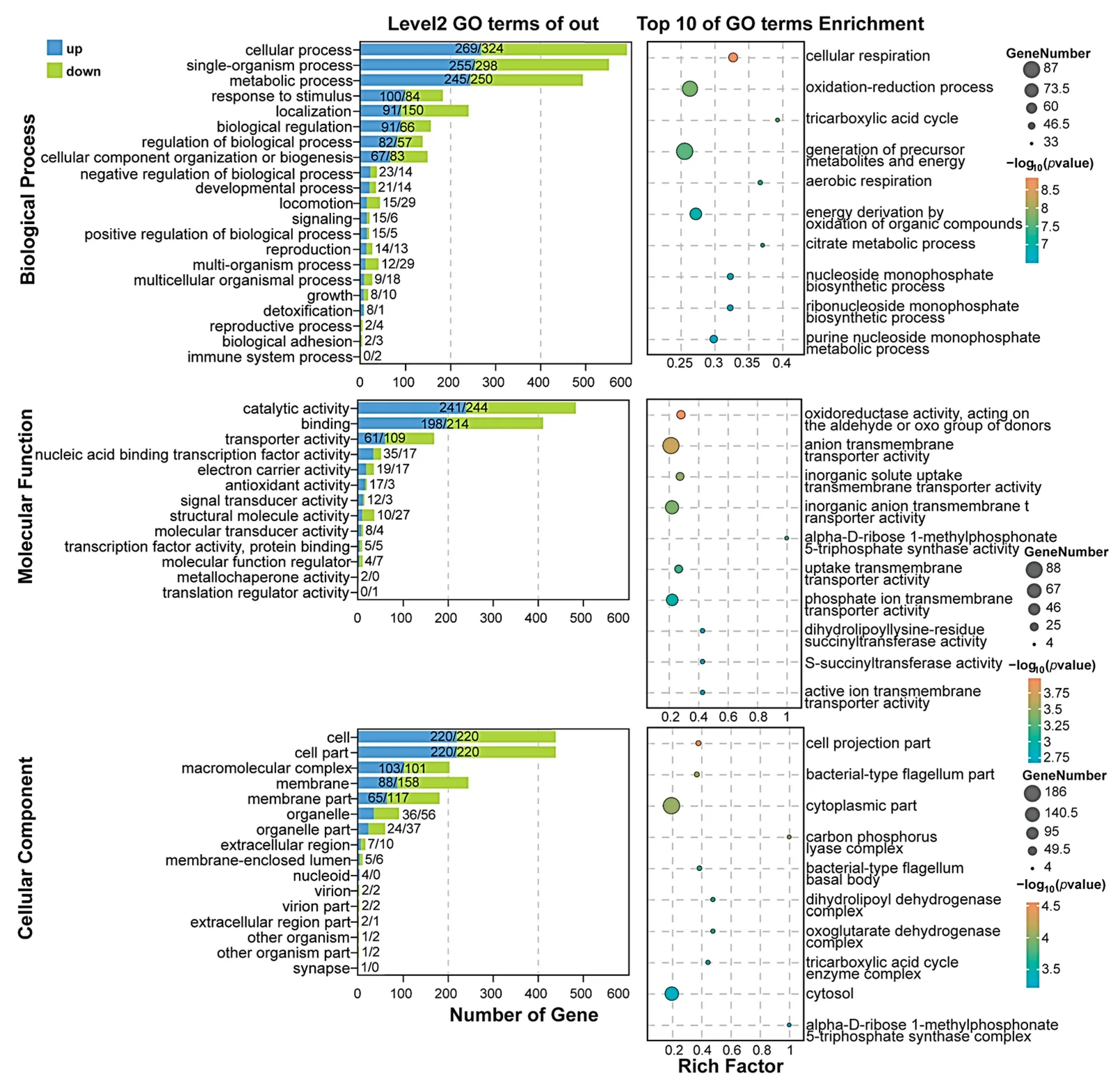
GO analysis of differentially expressed genes between groups ((DT02E vs. DT02T) vs. (DT07E vs. DT07T)) illustrated by data from DT07.

GO enrichment analysis was performed on the shared DEGs of (DT02E vs. DT02T) vs. (DT07E vs. DT07T), and DT07T vs. DT07E was chosen to illustrate the detailed data, as DT07 is the highly virulent strain and showed the larger transcriptional response to the medium change (3276 vs. 2084); the shared-DEG comparison described in Figure 3E,F includes both comparisons. The level 2 GO classification revealed that DEGs were distributed across all three functional categories. Among biological processes, the most enriched were cellular processes, single-organism processes, metabolic processes, and response to stimulus; among molecular functions, catalytic activity and binding were the most enriched categories; and among cellular components, cell, cell part, and membrane were the most enriched categories (Figure 4, left panel). The top 10 enrichment analysis (Figure 4, right panel) showed that biological processes were significantly enriched in cellular respiration, oxidation-reduction process, tricarboxylic acid cycle, aerobic respiration, and nucleotide metabolism, indicating that medium change primarily affected the expression of energy metabolism-related genes. In molecular function, oxidoreductase activity, anion transmembrane transporter activity, inorganic solute uptake transmembrane transport activity, and phosphate ion transmembrane transporter activity were significantly enriched. In cellular components, cellular projection part, bacterial-type flagellum part, cytoplasmic part, enzyme complexes tricarboxylic acid cycle enzyme complex, and carbon-phosphorus lyase complex were significantly enriched. These results indicate that culture conditions induce extensive transcriptional reprogramming, particularly affecting functions related to energy metabolism, substrate transport, and cell.

**Figure 5:**
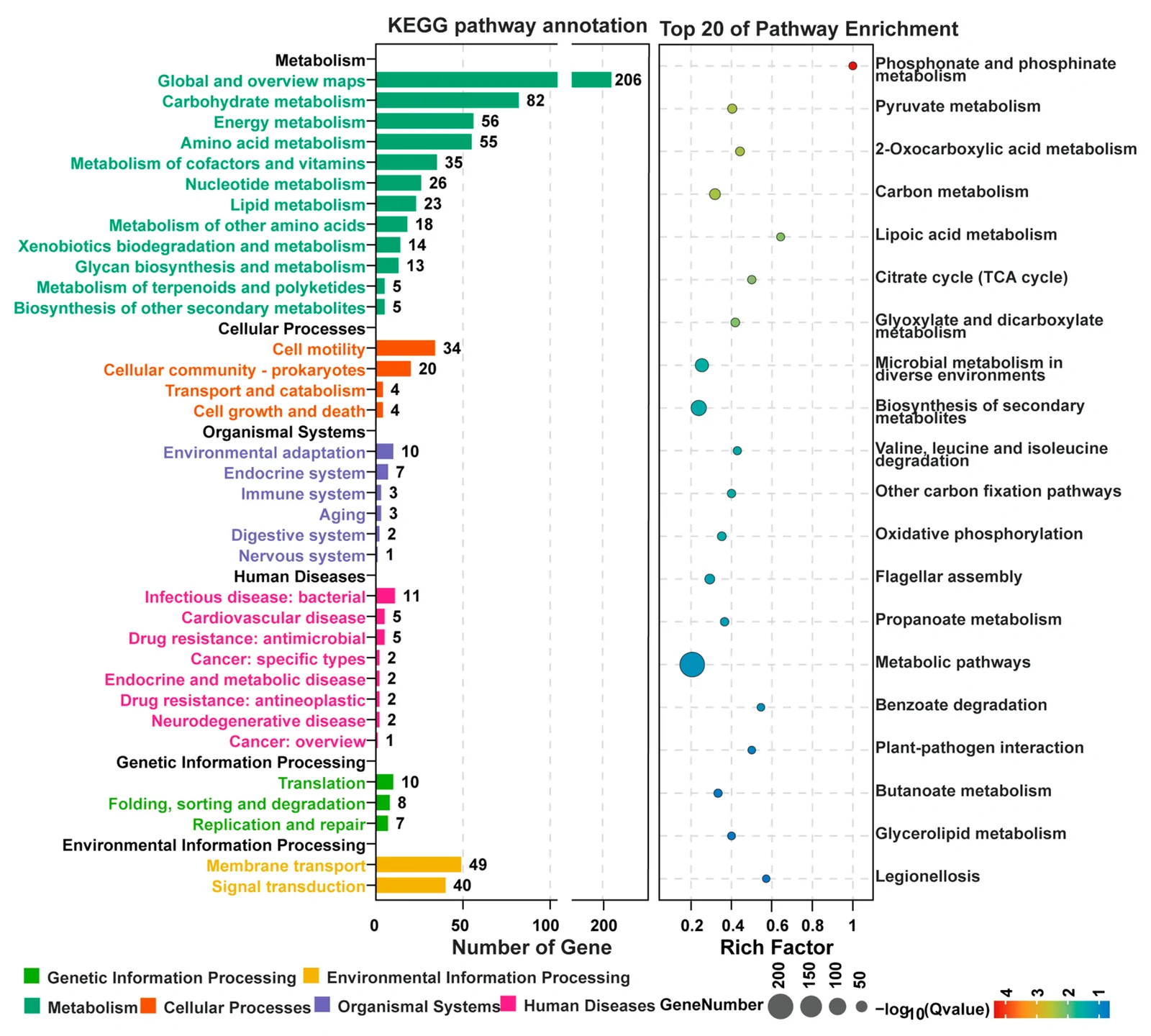
KEGG analysis of differentially expressed genes between groups ((DT02E vs. DT02T) vs. (DT07E vs. DT07T)) illustrated by data from DT07.

The KEGG annotation and enrichment analysis were performed to understand the DEG changes in response to culture conditions changing. KEGG pathway annotation showed that DEGs were mostly enriched in the metabolism category, with the most genes mapped to global and overview maps (206 genes), carbohydrate metabolism (82 genes), energy metabolism (56 genes), and amino acid metabolism (55 genes) (Figure 5, left panel). In addition, a notable number of DEGs were annotated to membrane transport (49 genes) and signal transduction (40 genes) under environmental information processing, as well as cell motility (34 genes) and cellular community-prokaryotes (20 genes) under cellular processes (Figure 5, left panel).

The top 20 enriched pathways were most enriched in carbon metabolism-related processes, including pyruvate metabolism, 2-oxocarboxylic acid metabolism, carbon metabolism, citrate cycle (TCA cycle), glyoxylate and dicarboxylate metabolism, oxidative phosphorylation, propanoate metabolism, and butanoate metabolism (Figure 5, right panel). Phosphonate and phosphonate metabolism showed the highest enrichment significance (smallest Q value). Furthermore, flagellar assembly was significantly enriched, corroborating the flagellum-related cellular component terms identified in the GO enrichment analysis. These results indicate that medium composition systematically alters the carbon utilization, energy production, ion transport, and cell motility pathways of *V. mediterranei*, with the enrichment of flagellar assembly.

**Figure 6:**
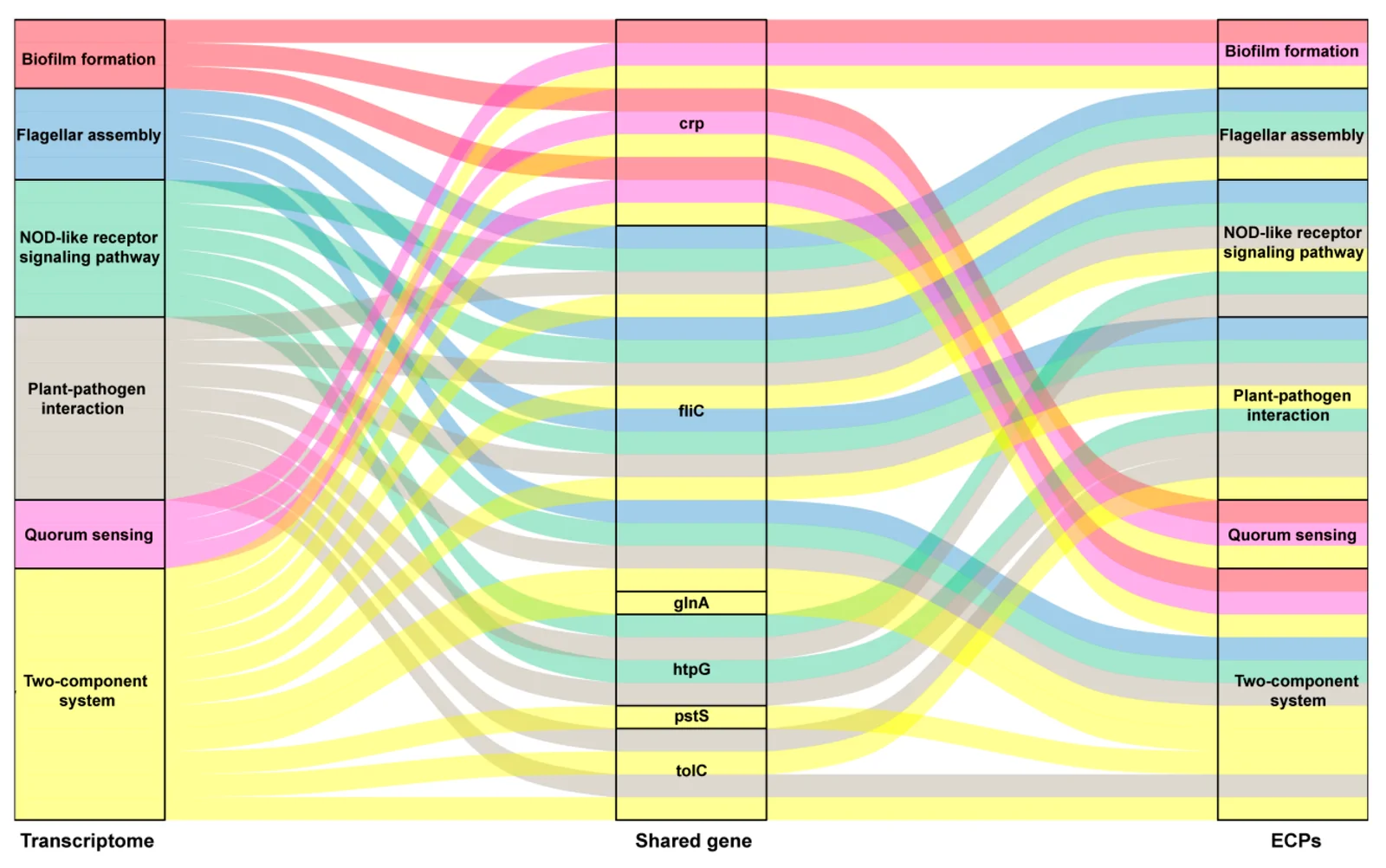
A Sankey diagram showing the integrated KEGG enrichment analysis in strain DT07, showing the six genes shared between the two omics layers (middle) and the enriched pathways in which their KEGG orthologs occur (left, transcriptome-enriched pathways; right, ECPs-enriched pathways). Ribbon width reflects the number of shared K numbers per pathway. The directions are given in Supplementary Table S5.

To determine whether the regulation of virulence-associated pathways by medium composition is consistent at both the transcriptomic and proteomic analyses, an integrated analysis was performed on the KEGG enrichment results of the transcriptome (DT07T vs. DT07E) and ECP proteome (DT07T vs. DT07E) (Figure 6). Using KO identifiers for cross-pathway gene comparison, the TCS (ko02020) exhibited the highest number of genes shared between the two omics, including *glnA* (K01915), *pstS* (K02040), *crp* (K10914), *fliC* (K02406), and *tolC* (K12340). The log2 fold change (TCBS/2216E) and the corresponding FDR values, together with the extracellular protein abundance ratios and the FDR values of the predicted candidate genes in both DT02 and DT07, are given in Supplementary Table S5. Each of these KO identifiers, except K04079, is carried by more than one genomic locus, and those loci did not always change in the same direction, so correspondence between the two layers is reported for the individual locus rather than for the gene name token. For the loci selected for comparisons, *glnA* and *crp* increased at both layers and *pstS* decreased at both layers, whereas *tolC* changed in the opposite direction in both strains, and only one of the seven flagellin loci followed the decrease at the protein level, indicating that extracellular protein abundance is not a single reflection of transcript abundance. The sixth shared gene shown in Figure 6, *htpG* (K04079), which belongs to the molecular-chaperone category rather than the TCS pathway, was up-regulated at both the transcript and the extracellular product layers in both strains (Supplementary Table S5). The *fliC* was identified across four virulence-associated pathways including TCS, flagellar assembly (ko02040), plant–pathogen interaction (ko04626), and NOD-like receptor signaling (ko04621). The latter two are organism-level KEGG annotation categories that also contain bacterial proteins; they are reported only as categories in which *fliC* is annotated and are not interpreted as evidence that the corresponding host signaling pathways are active in *V. mediterranei*. This indicates that both the transcription and extracellular abundance of flagellin are affected by the culture conditions. *tolC* was detected in the TCS and plant–pathogen interaction pathways, suggesting that the extracellular protein abundance of the outer membrane channel is modulated. Furthermore, *crp* appeared in the TCS, quorum sensing (ko02024), and biofilm formation (ko05111) pathways, implying that the carbon metabolism–virulence regulatory axis may be influenced by medium conditions. The results above suggested that TCS was prominently represented in the response to the change in culture environment, with *fliC*, *tolC*, and *crp* consistently detected at both omics layers, linking the transcriptional reprogramming to extracellular protein abundance (Figure 6).

In conclusion, in vivo infection assays demonstrated that all tested bacterial strains exhibited significantly elevated mortality when cultured in TCBS relative to 2216E, indicating that medium composition is a critical environmental determinant of bacterial pathogenicity. Transcriptomic and ECP proteomic analyses revealed that TCBS medium triggered extensive transcriptional reprogramming and extracellular protein remodeling, with convergent enrichment in the TCS, flagellar assembly, energy metabolism, and oxidative stress-related pathways. Cross-omics integration further identified *fliC*, *tolC*, and *crp* as genes detected at both omics layers, linking transcriptional regulation to extracellular protein abundance, with two-component signaling prominently represented among the pathways associated with motility, extracellular protein composition, and stress adaptation. These findings implicate that TCBS medium promotes *V. mediterranei* virulence through TCS-associated signaling, providing a framework for further validation connecting environmental cues to pathogenic potential in shellfish-associated *vibrios*.

## 4. Discussion

In this study, we demonstrated that the virulence of *Vibrio mediterranei* toward the razor clam *Sinonovacula constricta* is significantly affected by the culture medium, with strains in TCBS exhibiting higher pathogenicity than those in 2216E.

The integrated transcriptomic and proteomic study suggests that the composition of growth medium functions as a dominant environmental determinant of *V. mediterranei* pathogenicity. TCBS differs from 2216E in ways relevant to host–pathogen interactions, containing bile salts, citrate, and thiosulfate, and maintaining an alkaline pH of approximately 8.6 [20]. Bile salts have been shown to directly affect virulence gene expression in *Vibrio*. In *V. parahaemolyticus*, subinhibitory concentrations of sodium taurocholate induce a colonization-adapted state in which acute virulence determinants are suppressed while adhesion-related genes are upregulated [34] and dual-stress proteomic analyses have revealed bile-salt-dependent activation of secretion systems and metabolic remodeling [35]. In *V. cholerae*, bile salts influence the EnvZ/OmpR system, which in turn represses ToxR expression [17]. Additionally, the outer membrane channel TolC has been shown to be required for ToxR regulon expression [18]. Above all, these findings underscore that TCBS-specific components, particularly bile salts, function as global environmental cues that reprogram virulence regulatory networks across *Vibrio* species, demonstrating that growth medium composition is not merely a cultivation parameter but an active determinant of pathogenic potential.

Despite achieving similar high pathogenicity after TCBS culture, DT02 and DT07 adopt different regulatory strategies. DT07 produced substantially more DEGs (3276 vs. 2084) and exhibited a bias toward repression, whereas DT02 favored transcriptional activation (978 uniquely up-regulated genes vs. 2107 uniquely down-regulated in DT07). This suggests that the high-virulence strain maintains an elevated baseline expression of virulence functions that is passively exposed under TCBS stress, whereas the low-virulence strain requires active induction-a pattern consistent with genomic evidence that high-virulence strains harbor expanded repertoires of virulence-associated genes [12]. Despite these transcriptional differences, the extracellular proteome showed conserved interspecifically, with 169 up-regulated and 112 down-regulated proteins shared between strains, so that 281 of the 312 DEPs changed in the same direction in both strains. This divergence in regulatory strategy acting on a conserved functional output parallels observations in other pathogens [36] and may be further interpreted in light of recent phylogenomic evidence that *V. mediterranei* is divided into protective, pathogenic, and intermediate lineages with lineage-specific genomic architectures [37].

Integrated transcriptomic and proteomic data identified the TCS pathway as the category with the most genes differentially expressed at both mRNA and protein levels. This pattern was detected independently at each omics layer, reflecting concordance between the two layers rather than independent validation. The consistent detection of six genes at both the transcript and extracellular-protein levels provides concordance. The transcriptome showed differential expression of PhoB/PhoR, CpxA/CpxR, EnvZ/OmpR, KdpD/KdpE, ArcB/ArcA, and QseC/QseB, while the ECP proteome also returned TCS as a significantly enriched KEGG category. Several of these systems correspond to specific chemical features of TCBS. PhoB/PhoR, recently shown in *V. alginolyticus* to directly regulate virulence, with knockdown reducing fish mortality by 70% [38], responds to phosphate limitation. EnvZ/OmpR senses bile salts and osmotic shifts [17], and CpxA/CpxR may be activated by bile-salt-induced membrane perturbation [39]. The concurrent detection of PstS with PhoB/PhoR and TolC with EnvZ/OmpR is consistent with a model in which distinct TCBS components are sensed by parallel sensory modules whose outputs are associated with flagella assembly, extracellular protein composition, and central carbon metabolism. This conclusion remains inferential because no component of a TCS was manipulated here, and it mirrors the complex, multilayered roles of TCSs in *Vibrio* species [40,41].

The integrated transcriptomic and proteomic analysis identified three genes with cross-omics differential expression assigned to multiple KEGG pathways. *fliC* was the most among all cross-omics DEGs, appearing in four pathways. This reflects the dual function of FliC as the flagellar structural subunit required for motility and colonization [42], and as a TLR5 ligand that triggers NF-κB-mediated innate immune signaling [43,44], suggesting that TCBS-induced flagellar upregulation may simultaneously enhance colonization and contribute to host tissue damage. *tolC*, detected in the TCS and plant–pathogen interaction pathways, encodes the outer membrane channel of tripartite efflux and type I secretion systems. In *V. cholerae*, *tolC* deletion abolishes MARTX toxin secretion and attenuates ToxR regulon expression [18], consistent with a model in which medium-responsive TCS signaling is associated with altered *tolC* expression and with changes in the extracellular abundance of virulence-associated proteins, although the direction of change differed between the two omics layers and the three *tolC* loci of this annotation did not change in the same direction (Table S5) and no causal link was tested here. *crp*, detected in TCS, quorum sensing, and biofilm formation pathways, encodes the cAMP receptor protein, a global regulator that integrates carbon-source availability with virulence gene expression [30,45]. Recent evidence shows that post-translational acetylation of CRP Lys52 in *V. cholerae* expands its regulon to include additional virulence and sugar utilization genes [46], together with CRP-dependent modulation of biofilm formation through nitrate respiration control [47], positions CRP as a likely metabolic checkpoint linking TCBS carbon sources to virulence pathway activation.

The TCS-virulence associations reported here are correlative and require functional testing via targeted gene deletion and complementation. In the absence of such validation, we described the two-component system as prominently represented among the medium-responsive pathways, that is, as a candidate regulatory hub, rather than as a central hub or a TCS-centered regulatory network. Such an approach has been validated by recent work demonstrating that *phoR* silencing alone reduces *V. alginolyticus* virulence by 70% in a fish model [38]. Limitations of this study include the use of an in vitro medium that does not fully replicate the host environment, the reliance on pathway-level overlap rather than quantitative mRNA-protein correlation in the multi-omics integration, and the restriction of proteomic data to proteins recovered in the extracellular fraction. In addition, no cell-lysis control of cytoplasmic-marker analysis was performed, so the proteins detected in this fraction may be derived from active export, from outer-membrane vesicles, or from cell lysis; the differences reported here are described as changes in extracellular protein abundance rather than as changes in secretion. Despite these caveats, the consistent detection of five core genes across two independent omics platforms provides cross-platform validation, partially offsetting the lack of qRT-PCR confirmation. In conclusion, our findings support a model in which TCBS medium engages multiple TCSs associated with transcriptional reprogramming and remodeling of the extracellular protein complement, together with changes in motility (*fliC*), the outer-membrane channel (*tolC*), and metabolic adaptation (*crp*). This model is inferred from correlative omics evidence rather than from a exacted demonstration of TCS-mediated mechanism, and these candidates remain to be tested by targeted deletion and complementation. An important consideration is that bivalves lack a hepatobiliary system and do not produce bile salts, the canonical inducers of the ToxR/ToxS virulence cascades in vertebrate pathogenic *vibrios* [13,14]. Thus, while bile salts in TCBS may artificially mimic vertebrate host signals, nutrient and osmolarity sensing in the bile-salt-free bivalve hemocoel could similarly potentiate virulence through TCS pathways [48]. Future work should prioritize constructing defined TCS mutants in *V. mediterranei*, establishing in vivo infection models that incorporate host immune analysis, constructing paired quantitative transcript-protein correlation across the full proteome, and surveying TCS network conservation across the expanding *V. mediterranei* species complex, including its recently identified non-pathogenic and protective lineages.

## Data Availability

The transcriptomic raw data (accession no. CRA050264) and proteomic data (accession no. OMIX021412-02) have been deposited in the Genome Sequence Archive (GSA) and the Open Archive for Miscellaneous Data (OMIX), separately, under the same sample project (accession no. PRJCA074606) at the National Genomic Data Center (CNCB) and are publicly accessible at https://ngdc.cncb.ac.cn/.

## Supplementary Materials

The following supporting information can be downloaded at: https://www.mdpi.com/article/10.3390/vetsci13101069/s1, Figure S1: Kaplan–Meier survival curve of razor clam juveniles exposed to bacteria cultured in TCBS, 2216E or sterile seawater (control); Table S1: Transcriptome sequencing and alignment statistics for the four groups (DT02E, DT02T, DT07E, DT07T; three replicates each). Genes were counted as detected when at least one read was mapped, against a reference set of 6,178 loci; Table S2: GO enrichment results for the concordantly down-regulated shared DEPs (n = 112, 43 with GO annotation; background n = 419; hypergeometric test with BH correction). The 297 tested terms are listed with GO ID, ontology, Ratio, *P* value, FDR; Table S3: GO enrichment results for the concordantly up-regulated shared DEPs (n = 169, 137 with GO annotation; background n = 419; hypergeometric test with BH correction); Table S4: Differentially expressed extracellular proteins (DEPs) of strains DT02 and DT07 cultured in TCBS versus 2216E (fold change > 1.3 or < 0.77, BH-adjusted *P* < 0.05); Table S5: Transcriptomic (sheet 1) and extracellular protein (sheet 2) statistics for the six candidate genes shared between the two omics layers.; Table S6: Cumulative 36 h mortality of razor clam juveniles recorded in each container. Each condition comprised three containers of 30 juveniles; the container was the unit of statistical analysis. “Control” means juveniles immerged in sterile seawater without bacteria.

## Abbreviations

The following abbreviations are used in this manuscript:

BCA	Bicinchoninic acid
CRP	cAMP receptor protein
DEGs	Differentially expressed genes
DEPs	Differentially expressed proteins
ECPs	Extracellular products
FDR	False discovery rate
GO	Gene Ontology
KEGG	Kyoto Encyclopedia of Genes and Genomes
KO	KEGG Orthology
LC-MS/MS	Liquid chromatography-tandem mass spectrometry
LFQ	Label-free quantification
MSH	Melanocyte-stimulating hormone
PCA	Principal component analysis
RTX	Repeats-in-toxin
SOD	Superoxide dismutase
TCA	Tricarboxylic acid cycle
TCBS	Thiosulfate citrate bile salts sucrose
TCP	Toxin-coregulated pilus
TCS	Two-component system
UPLC	Ultra-performance liquid chromatography
