# Supplementary material for: Transcriptomic and Extracellular Proteomic Analyses Implicate Two-Component Signaling in Medium-Dependent Virulence in Vibrio mediterranei
Source: Vet Sci. 2026 Oct 10;13(10):1069. doi: 10.3390/vetsci13101069 (PMC13709680; doi:10.3390/vetsci13101069)
Supplement: Supplementary file 1 [file vetsci-13-01069-s001.zip › Supplementary Table S1.pdf]

Supplements

Table S1 Transcriptomic analysis results

Sample	Raw reads	Clean reads (%)	Reads after rRNA removal	Uniquely mapped (%)	Mapping ratio (%)	Known genes detected (%)
**DT02E-1**	19738320	19224492 (97.40)	19149552	17579365 (91.80)	93.15	4966 (80.38)
**DT02E-2**	19772804	19092666 (96.56)	18946156	17011318 (89.79)	91.16	4895 (79.23)
**DT02E-3**	19447362	18757068 (96.45)	18538562	16718883 (90.18)	91.71	4918 (79.61)
**DT02T-1**	18922284	18373374 (97.10)	17860798	15111444 (84.61)	87.03	4886 (79.09)
**DT02T-2**	19734252	19239536 (97.49)	18818426	16056291 (85.32)	87.74	4857 (78.62)
**DT02T-3**	19525356	19065892 (97.65)	18644220	15964552 (85.63)	88.14	4845 (78.42)
**DT07E-1**	18930906	18843710 (99.54)	16398378	15537625 (94.75)	98.01	5326 (86.21)
**DT07E-2**	18142684	18046644 (99.47)	17123682	16187142 (94.53)	97.91	5339 (86.42)
**DT07E-3**	19529830	19437506 (99.53)	18405716	17403876 (94.56)	97.93	5328 (86.24)
**DT07T-1**	16702318	16619922 (99.51)	15930544	15209566 (95.47)	98.86	5171 (83.70)
**DT07T-2**	15642590	15570282 (99.54)	15074972	14394365 (95.49)	98.94	5218 (84.46)
**DT07T-3**	19192996	19088854 (99.46)	18469166	17658909 (95.61)	98.96	5196 (84.10)

Note: reference genes: 6178. Gene detected were counted as known genes with at least one mapped read. Value in parentheses is percentage of reference genes.
